# Venous-Only Approach for Transcatheter Patent Ductus Arteriosus Closure in Infants: Is It Time for Change?

**DOI:** 10.1016/j.jscai.2025.103735

**Published:** 2025-07-23

**Authors:** Shyam C. Harinarayanan, Stephan Wu, Yousef Arar, Thomas M. Zellers, Surendranath R. Veeram Reddy, Abhay A. Divekar

**Affiliations:** a Department of Pediatrics, UT Southwestern Medical Center, Dallas, Texas; b Division of Pediatric Cardiology, Children’s Medical Center Dallas, Heart Center, Dallas, Texas

**Keywords:** arterial complications, outcomes, transcatheter patent ductus arteriosus closure

## Abstract

**Background:**

The standard approach for transcatheter closure of patent ductus arteriosus (TC-PDA) requires arterial access and is associated with the risk of arterial injury, a metric tracked by national quality improvement registries. Venous-only TC-PDA in premature infants is performed successfully without arterial access. It was hypothesized that PDA closure in infants could be performed safely and effectively without arterial access.

**Methods:**

This is a single-center, retrospective, institutional review board–approved study. All infants weighing 2 to 10 kg who underwent TC-PDA closure between January 2019 and December 2024 were included in the study. Patients who underwent concurrent procedures and those with complex heart disease were excluded from the study. TC-PDA was performed using the standard approach or venous-only approach at the discretion of the attending cardiologist.

**Results:**

In total, 150 patients underwent TC-PDA: 59 patients (19 male) underwent closure using the standard approach and 91 patients (35 male) using the venous-only approach. There was no difference in the minimum patent ductus arteriosus diameter. There was no pulse loss in the venous-only cohort; 1 patient (1.7%) treated with the standard approach had clinical symptomatic arterial injury requiring therapy. The venous-only cohort was younger, weighed less, and had lower radiation exposure, contrast use, shorter total procedure, and total sheath time. There was no difference in other adverse procedural outcomes.

**Conclusions:**

Venous-only approach for TC-PDA closure in infants is as effective and efficient as the standard approach. The added advantage of eliminating arterial injury increases the safety for TC-PDA in small infants.

## Introduction

Transcatheter closure of patent ductus arteriosus (TC-PDA) is standard of care for all age groups and increasingly performed in premature neonates.^1^ The standard approach for TC-PDA closure except in premature neonates includes obtaining femoral arterial and venous access. Arterial access is used to perform baseline aortography to define ductal morphology and guide device selection. Following device closure (from a transvenous approach), aortography is repeated to evaluate device position, aortic obstruction, and residual shunting; in a small proportion, the patent ductus arteriosus (PDA) is closed from the arterial approach. Leading to and following the US Food and Drug Administration approval (2019) of the Amplatzer Piccolo device (Abbott Structural Heart), TC-PDA in premature infants is now routinely offered and successfully performed as an alternative to surgical closure.^1^ A prerequisite for successful premature PDA closure is the need for avoidance of arterial access, limited angiography, and extensive use of echocardiography to guide all aspects of closure, especially after device deployment and prior to release; as a direct consequence, operator expertise for venous-only approach for TC-PDA closure has significantly increased.

In an informal national survey of 14 high-volume institutions in the United States routinely offering TC-PDA closure in the premature neonates, the standard approach is still used in small infants (2-10 kg). The reasons cited include concerns for catheter-induced ductal spasm, inadequate angiographic evaluation with venous-only access, perceived limitations of echocardiography to adequately define ductal dimensions, and the time and resources required for echocardiographic guidance impacting total procedure time. This same population of small infants, however, is at risk of femoral arterial injury during TC-PDA closure, a quality metric tracked by national registries.^2^ This discrepancy in approach between similar procedures prompted us to retrospectively review our experience with TC-PDA closure in infants’ weighing 2-10 kg and review the published literature.

## Materials and methods

This is a single-center, retrospective, institutional review board–approved study with waiver of consent. All infants, ≥2 and ≤10 kg referred to the pediatric cardiac catheterization laboratory for standard of care TC-PDA closure between January 1, 2019, and December 31, 2024 were included for analysis if the PDA device was delivered transvenous (antegrade). Patients who underwent concomitant interventional procedures, those with preprocedural concern for aortic arch hypoplasia or coarctation, and those with associated significant congenital heart disease were excluded from the analysis.

Patients were identified from the cardiac catheterization database. Electronic medical records, catheterization, and echocardiographic reports were reviewed. Demographic characteristics; catheterization-related metrics (radiation metrics, total contrast, total procedure time defined as room in to room out time, total sheath time [sheath in to sheath out], PDA dimensions [minimum PDA dimension and PDA length] and morphology); and outcome metrics including residual shunting, pulmonary artery or aortic obstruction, tricuspid valve injury, pulse loss requiring treatment, number of devices used, type of device used, trainee participation, device embolization, and number of unsuccessful closures were recorded.

### TC-PDA closure

Starting at the end of 2021, during our catheterization laboratory quality improvement meeting, a venous-only approach for TC-PDA closure in infants weighing ≤10 kg was proposed by the senior author (A.D.). At our institution, there were 2 approaches for TC-PDA closure in infants; all aspects of TC-PDA closure (including choice of device) were at the discretion of the attending interventional cardiologist. In the first approach, the standard approach, an appropriately sized sheath was placed in the artery and vein using ultrasound guidance. Aortic angiography is performed before advancing any catheter into the pulmonary artery for hemodynamic assessment (to prevent ductal spasm). The patient then undergoes antegrade TC-PDA closure (device delivered from the venous side) using standard catheterization techniques. The adequacy of device closure was determined by angiography. In the second approach, the venous-only approach (Central Illustration), the patient undergoes echocardiography (performed by a technologist with immediate availability of attending echocardiographer if required) after induction of anesthesia to assess PDA morphology and size. Echocardiographic minimum PDA dimension obtained from multiple views with active guidance from the attending interventional cardiologist is used to select device size, unless the subsequent angiographic measurement is larger. The PDA length and morphology are determined by angiography. A venous sheath is placed under ultrasound guidance and the PDA crossed antegrade. PDA angiography is performed through the long sheath used to deliver the device. After deployment, with the device still attached, angiography is performed through the delivery sheath to assess the device position on the pulmonary artery side. Echocardiography is used to assess residual shunting, arch, and left pulmonary artery (LPA) obstruction before and after releasing the device (Central Illustration). Following release, the tricuspid valve is assessed for inadvertent injury. Follow-up care and imaging were at the discretion of the attending cardiologist. Only clinically apparent arterial injury is investigated and treated (specifically routine ultrasound evaluation for access site complications is not performed).

**Central Illustration:**
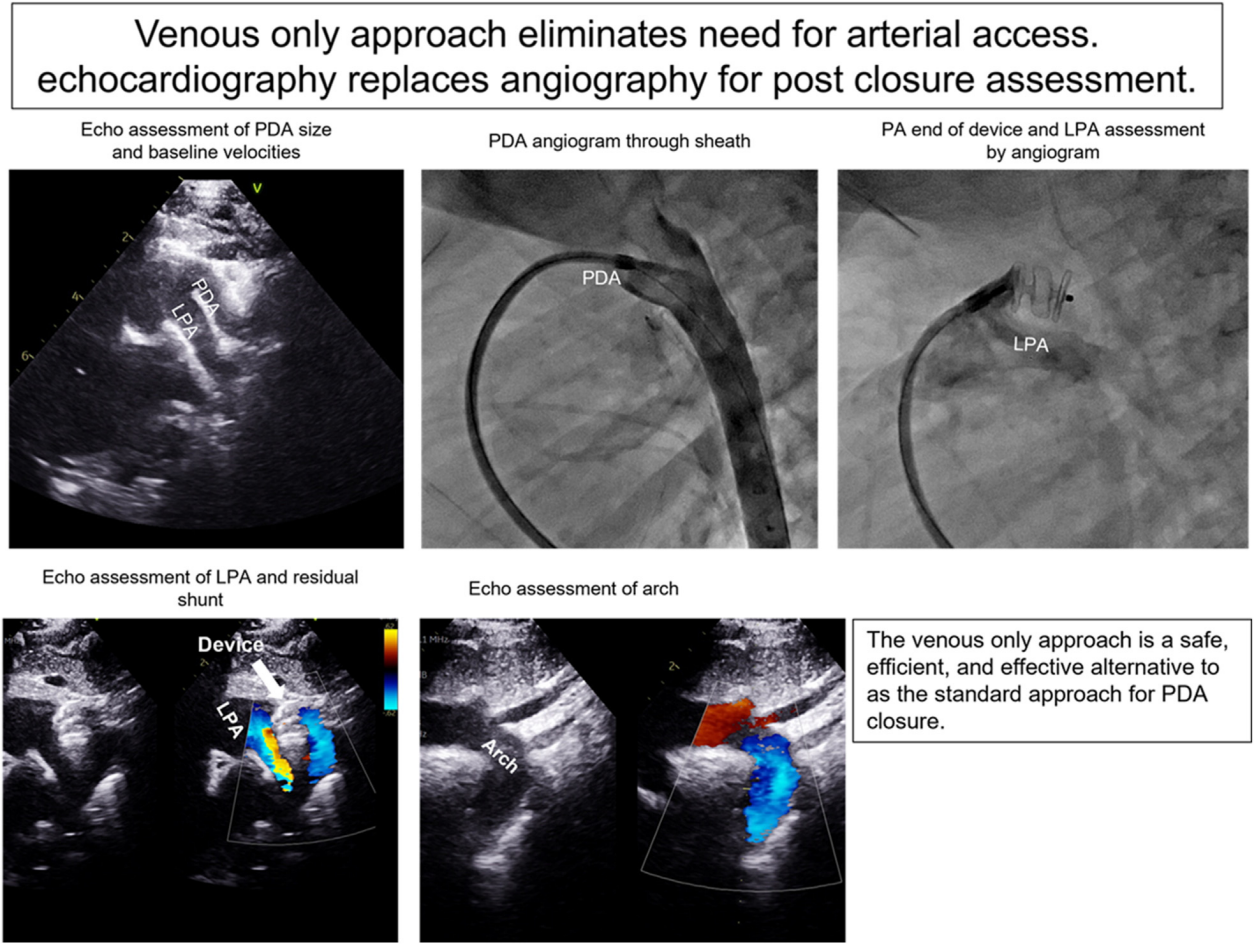
**Echocardiography-guided transcatheter patent ductus arteriosus (PDA) closure eliminates the need for arterial access.** After device placement, echocardiography replaces angiography for assessing adequacy of closure and to confirm that there is no obstruction to the left pulmonary artery (LPA) or aortic arch before and after release of the device.

Significant LPA obstruction was defined as a continuous wave Doppler velocity ≥2.5 m/s with antegrade diastolic flow or any LPA stenosis requiring intervention. Aortic arch obstruction was defined as a continuous wave Doppler velocity of >1.5 m/s with antegrade diastolic flow or any obstruction requiring intervention.

Statistical analysis was performed using SPSS Statistics version 27 (IBM Corp). Data are reported as median (IQR) for continuous variables and count (percentage of total) for categorical variables. Statistics were evaluated using nonparametric Mann-Whitney *U* test, with statistical significance set at *P* < .05.

## Results

**Figure 1:**
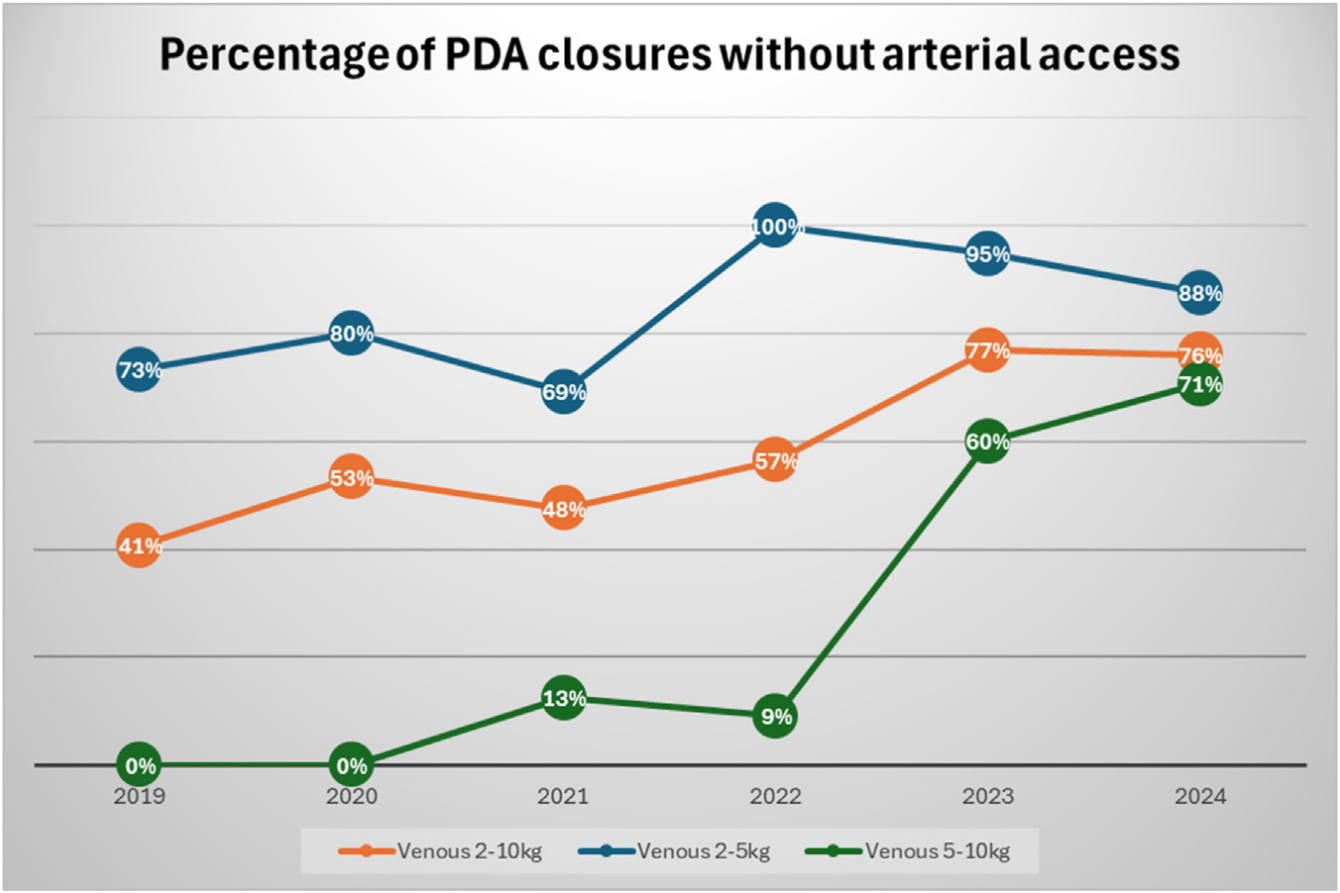
**Percentage of PDA closdures without arterial access in infants 2-10 kg from 2019-2024.** Following discussion to consider venous-only approach for transcatheter PDA closure in infants 2-10 kg starting in late 2021, there was an increase in number of venous-only procedures in all weight categories. PDA, patent ductus arteriosus.

Between January 2019 and December 2024, 150 patients weighing ≥2 kg and ≤10 kg underwent TC-PDA closure: the standard approach was used in 59 patients (19 male), and 91 patients (35 male) underwent the venous-only approach. Demographic characteristics, procedural, and outcome metrics are summarized (Table 1). In the venous-only group, patients were younger and weighed less when compared with those who underwent TC-PDA closure using the standard approach. All patients who underwent TC-PDA using the standard approach had a 4F Terumo Pinnacle Introducer sheath (Terumo Medical) placed in the femoral artery. There was no difference in the minimum diameter of the PDA between the groups; the PDA was longer in the venous-only cohort. The venous-only cohort had lower radiation exposure, lower contrast use, and shorter total procedure time and total sheath time. There was no pulse loss in patients who underwent venous-only approach; 1 patient (1.7%) in the standard approach cohort had clinically symptomatic arterial injury requiring therapy (confirmed by ultrasound) and was successfully treated with intravenous heparin, followed by low molecular weight heparin for 5 days, with resolution of thrombosis and improved lower extremity perfusion. Follow-up ultrasound showed patent vessel with minimal stenosis. There was no difference in adverse outcomes including pulmonary artery or aortic obstruction, significant new tricuspid regurgitation, residual shunt, maximum number of devices attempted in each patient, trainee participation, device embolization, or unsuccessful procedures between the 2 groups (Table 1). Subgroup analysis in patients weighing 2 to 5 kg and >5 kg showed similar results (Table 2, Table 3, respectively). Starting in late 2021, there was gradual increase in adoption of venous-only TC-PDA closure in young infants weighing ≥2 kg (Figure 1) as the benefits and efficiency of the approach became more evident. Among the entire cohort, follow-up echocardiograms were available for review in 86% of the patients ≥1 day following the procedure, in 75% of patients for ≥7 days following the procedure, and in 46% of patients for ≥30 days following the procedure. Two patients (3.4%) in the standard approach group had a significant shunt associated with device embolization, with both requiring surgical device removal. One patient (1.1%) in the venous-only group had device embolization requiring transcatheter retrieval and spontaneous closure of the PDA.

**Table 1:** Demographic characteristics, procedural, and outcome metrics for all patients (2-10 kg).

Characteristics	Standard approach (n = 59)	Venous-only (n = 91)	*P*
Sex, n			.437
Male	19	35	
Female	40	56	
Age, d	252 (168-371)	93 (63-211)	<.001
Weight, kg	7.1 (5.3-8.7)	3.2 (2.6-5.8)	<.001
Height, cm	67 (60-74)	49 (45-61)	<.001
PDA length, mm	11. (8.6-13.1)	12.9 (10.9-15)	.001
Minimum size of PDA, mm	3.1 (2.3-4.3)	3 (2.2.5-3.7)	.503
Fluoroscopy time, min	12 (8.9-17.4)	8.1 (5.4-13)	<.001
Radiation exposure DAP(μGy∗m^2^)/kg)	18.8 (12.8-30)	11.2 (6.6-21.3)	<.001
Total contrast, mL	4.2 (3.3-5.6)	1.8 (1.2-2.9)	<.001
Total procedure time (room in − room out), min	132 (115-161)	115 (97-133)	<.001
Sheath time, min	63 (52-88)	48 (38-63)	<.001
Trainee present			.303
Yes	46 (78)	64 (70.3)	
No	13 (22)	27 (29.7)	
Device type			<.001
ADO	34 (57.6)	9 (9.9)	
AVP	25 (42.4)	61 (67)	
KA	0 (0)	5 (5.5)	
MVP	0 (0)	10 (11)	
Piccolo	0 (0)	6 (6.6)	
PDA morphology			<.001
Type A	39 (66.1)	23 (25.3)	
Type C	5 (8.5)	17 (18.7)	
Type D	3 (5.1)	3 (3.3)	
Type E	12 (20.3)	48 (52.7)	
LPA obstruction	0 (0)	0 (0)	.421
Aortic obstruction	0 (0)	0 (0)	>.99
Tricuspid regurgitation ≥moderate	0 (0)	0 (0)	>.99
Unsuccessful procedure	2 (3.4)	1 (1.1)	.329
Embolization	2 (3.4)	1 (1.1)	.329
Pulse loss	1 (1.7)	0 (0)	.214
Residual shunt ≥moderate	2 (3.4)	0 (0)	.078
No. of devices used			.009
1	41 (69.5)	79 (86.8)	
2	10 (16.9)	7 (7.7)	
3	5 (8.5)	4 (4.4)	
4	3 (5.1)	1 (1.1)	

Values are median (IQR) or n (%).

ADO, Amplatzer Ductal Occluder I; AVP, Amplatzer Vascular Plug II; DAP, dose area product; LPA, left pulmonary artery; MVP, Medtronic microvascular plug; KA, microplug set; Piccolo, Amplatzer Piccolo Occluder.

**Table 2:** Demographic characteristics, procedural, and outcome metrics for patients 2-5 kg.

Characteristics	Standard approach (n = 12)	Venous-only (n = 65)	*P*
Sex, n			.208
Male	3	29	
Female	9	36	
Age, d	167.5 (93.3-216.5)	73 (54.5-104)	.003
Weight, kg	4.5 (4.1-4.8)	2.9 (2.4-3.5)	<.001
Height, cm	57 (54.3-60.8)	46 (44-50)	<.001
PDA length, mm	10.4 (8.3-13.7)	13 (11-15.2)	.033
Minimum size of PDA, mm	2.9 (2.2-4.2)	3 (2.4-3.9)	.866
Fluoroscopy time, min	14.5 (9.5-25.2)	8.1 (5.2-12.7)	.003
Radiation exposure DAP(μGy∗m^2^)/kg)	20 (15.4-36.7)	12.3 (6.9-23.7)	.039
Total contrast, mL	4.7 (3.4-5.9)	2 (1.3-3.1)	<.001
Total procedure time (room in − room out), min	140 (121.3-167.8)	115 (96-132.5)	.003
Sheath time, min	71.5 (59.8-102.8)	51 (38--63.5)	.003
Trainee present			.861
Yes	8 (66.7)	45 (69.2)	
No	4 (33.3)	20 (30.8)	
Device type			<.001
ADO	7 (58.3)	3 (4.6)	
AVP	5 (41.7)	41 (63.1)	
KA	0 (0)	5 (7.7)	
MVP	0 (0)	10 (15.4)	
Piccolo	0 (0)	6 (9.2)	
PDA morphology			<.001
Type A	8 (66.7)	10 (15.4)	
Type C	1 (8.3)	10 (15.4)	
Type D	1 (8.3)	2 (3.1)	
Type E	2 (16.7)	43 (66.2)	
LPA obstruction	0 (0)	0 (0)	.667
Aortic obstruction	0 (0)	0 (0)	>.99
Tricuspid regurgitation ≥moderate	0 (0)	0 (0)	>.99
Unsuccessful procedure	0 (0)	1 (1.5)	.667
Embolization	0 (0)	1 (1.5)	.667
Pulse loss	0 (0)	0 (0)	>.99
Residual shunt ≥moderate	0 (0)	0 (0)	>.99
No. of devices used			.743
1	11 (91.7)	57 (87.7)	
2	0 (0)	5 (7.7)	
3	1 (8.3)	3 (4.6)	

Values are median (IQR) or n (%).

ADO, Amplatzer Ductal Occluder I; AVP, Amplatzer Vascular Plug II; DAP, dose area product; LPA, left pulmonary artery; MVP, Medtronic microvascular plug; KA, microplug set; Piccolo, Amplatzer Piccolo Occluder.

**Table 3:** Demographic characteristics, procedural, and outcome metrics for patients 5-10 kg.

Characteristics	Standard approach (n = 47)	Venous-only (n = 26)	*P*
Sex, n			.332
Male	16	6	
Female	31	20	
Age, d	308 (213-387)	238.5 (209.25-312)	.3
Weight, kg	7.7 (6.2-9)	7 (6-8.6)	.256
Height, cm	69.5 (65-75)	64 (60.5-69.3)	.006
PDA length, mm	11 (8.6-13.1)	12 (10.2-15.1)	.108
Minimum size of PDA, mm	3.2 (2.3-4.5)	3 (2.6-3.5)	.5
Fluoroscopy time, min	11.7 (8.8-16.5)	8 (6-13.4)	.003
Radiation exposure DAP(μGy∗m^2^)/kg)	18.6 (12.5-26.2)	9.7 (6.1-15.9)	<.001
Total contrast, mL	4.2 (3.3-5.6)	1.38 (1.1-2)	<.001
Total procedure time (room in − room out), min	129 (110-156)	115 (99-138.3)	.041
Sheath time, min	63 (51-82)	45 (37.5-62.3)	<.001
Trainee present			.445
Yes	38 (80.9)	19 (73.1)	
No	9 (19.1)	7 (26.9)	
Device type			.005
ADO	27 (57.4)	6 (23.1)	
AVP	20 (42.6)	20 (76.9)	
PDA morphology			.375
Type A	31 (66)	13 (50)	
Type C	4 (8.5)	7 (26.9)	
Type D	2 (4.3)	1 (3.8)	
Type E	10 (21.3)	5 (19.2)	
LPA obstruction	0 (0)	0 (0)	>.99
Aortic obstruction	0 (0)	0 (0)	>.99
Tricuspid regurgitation ≥moderate	0 (0)	0 (0)	>.99
Unsuccessful procedure	2 (4.3)	0 (0)	.289
Embolization	2 (4.3)	0 (0)	.289
Pulse loss	1 (2.1)	0 (0)	.457
Residual shunt ≥moderate	2 (4.3)	0 (0)	.289
No. of devices used			.074
1	30 (63.8)	22 (84.6)	
2	10 (21.3)	2 (7.7)	
3	4 (8.5)	1 (3.8)	
4	3 (6.4)	1 (3.8)	

Values are median (IQR) or n (%).

ADO, Amplatzer Ductal Occluder I; AVP, Amplatzer Vascular Plug II; DAP, dose area product; LPA, left pulmonary artery.

## Discussion

In this retrospective study, the venous-only approach for TC-PDA closure in infants was found to be safe, effective, and efficient when compared with the standard approach. Importantly, avoidance of arterial access eliminated the risk of arterial injury in the venous-only group; the rate of pulse loss in the standard approach group (1.7%) was lower than that reported in the literature.^2^, ^3^, ^4^, ^5^, ^6^, ^7^, ^8^ All patients in our study underwent closure using the standard approach had a 4F Terumo Pinnacle Introducer sheath placed in the femoral artery. Using a sheath such as the 4F Prelude Ideal Hydrophilic Sheath Introducer (Merit Medical Systems) or the 3.3F PediaVascular Super Sheath Introducer Sheath (PediaVascular), which have the same smallest outer diameter, may further help reduce the risk and should be considered.^9^ Glatz et al^2^ have reported a considerable risk of arterial injury in infants; the highest risk was for small infants (<4 kg [23.4%]; 4-6 kg [14.8%]). Alexander et al^8^ have shown that even with careful ultrasound-guided vascular access, infants younger than 6 months had 18.5% prevalence of pulse loss. Abu Hazeem et al^7^ reported that 3 of the 8 patients weighing 2 to 4 kg developed arterial thrombosis while undergoing TC-PDA closure using the standard approach. In this study, in the standard approach cohort, 1 of the 59 (1.7%) patients had clinical evidence of arterial injury manifesting as pulse loss needing medical therapy and resolved with anticoagulation therapy. This patient weighed 7.1 kg; the procedural metrics for this patient (total procedural time, 159 minutes; total sheath time, 68 minutes; radiation exposure, dose area product/kg 20.8 μGy∗m^2^) were not outside the range for the rest of the cohort (Table 1, Table 3).

In this study, using the venous-only approach was as effective as the standard approach with no difference in closures success or complication rates. In retrospective studies by Deraz et al^3^ (320 patients), Baykan et al^4^ (101 patients), and Garg et al^5^ (179 patients), the procedural outcomes following TC-PDA closure were comparable between the standard and the venous-only approach. While the patients in all these studies were older and weighed more than patients in our study, there was still a significant rate of arterial injury (5.4%-9.8%).^6^ While the procedural outcomes were comparable, 16% of patients with arterial access developed vascular injury. In this study, 3 patients had unsuccessful procedures; 2 patients in the standard approach had device embolization and required surgical retrieval and PDA closure; 1 patient in the venous-only group had device embolization, underwent successful transcatheter retrieval, and had spontaneous PDA closure. No patients in the venous-only group required conversion to the standard approach. Finally, the venous-only approach was as efficient as the standard approach without compromising procedural success; in fact, the venous-only group had lower radiation exposure, lower contrast use, and shorter procedure times.

Several specific observations from the study warrant mention. Ductal spasm can certainly occur whenever the PDA is crossed prior to angiography and can result in erroneous measurements and therefore incorrect device selection. Therefore, when performing venous-only TC-PDA closure, it is critical to perform baseline echocardiogram for ductal dimensions prior to any catheter manipulation across the PDA. When the angiographic dimension is smaller than the echocardiographic dimension, the possibility of ductal spasm should be considered when choosing the device. In this study, the incidence of residual shunt, device embolization, and LPA or aortic obstruction did not differ between the 2 groups, supporting the notion that clinically problematic ductal spasm did not affect procedural outcomes. Early in the experience, it was recognized that angiography through a catheter was not suitable for assessment of majority of the PDAs. However, an angiogram performed through the long sheath over a wire allowed for precise positioning of the sheath to optimize opacification of the PDA without losing access across the PDA. The total procedure time and total sheath time was shorter for the venous-only approach (echocardiography did not add to procedural time), and the metrics of radiation exposure and contrast use were lower for the venous-only approach. These observations are in keeping with the published literature and suggest that the perceived limitations of the venous-only approach do not interfere with successful procedures.^3^, ^4^, ^5^, ^6^, ^7^ Similar to the experience with premature neonatal PDA closure, there are institutional variations in the approach to echocardiography. At our institution, a technologist performs the echocardiogram after the patient is anesthetized and is positioned (including arms position) but before the sterile preparation. This allows optimizing neck extension to obtain adequate acoustic windows. The echocardiogram is focused and targeted toward specifics of TC-PDA closure at the direction of the attending interventional cardiologist. The minimum dimension of the PDA is measured from a short-axis view and the suprasternal notch view (3-vessel view). Color Doppler is incorporated to avoid overestimation or underestimation of the size. Other factors incorporated in interpreting appropriateness of measurements include magnitude of shunt (left heart dilation) and restriction across the ductus. The device size is based on the larger of the echocardiographic or angiographic measurement. Institutions performing premature neonatal PDA closure have optimized these technical requirements at their institutions and can easily adapt their protocols to small infant PDA closure.

Based on this study and the published literature, the data support that TC-PDA closure with a venous-only approach is as safe, effective, and efficient as the standard approach. Minimizing the use of arterial access for routine PDA closures provides tangible reduction in procedural risk profile in addition to other benefits including reduced contrast and radiation exposure. In this study, infants with concern for arch hypoplasia were excluded for venous-only TC-PDA closure. TC-PDA closure in this group may still benefit from arterial access for hemodynamic and angiographic assessment of the arch following device placement. In patients with small PDAs, where crossing of the PDA from the venous side is anticipated to be challenging, the standard approach, including closure from the arterial side may be preferable and should be at the discretion of the operator. The lessons learned and experience gained from TC-PDA closure in premature infants can be immediately implemented in small infants who are at the greatest risk for arterial injury.

## Conclusion

Our study findings add to the increasing evidence that TC-PDA closure in infants using venous-only approach can be performed effectively, efficiently, and with improved safety compared with those of traditional standard techniques.

In addition to this being a retrospective study, there are several limitations. There is a selection bias; TC-PDA closure strategy was at discretion of the attending cardiologist, and randomization was not performed. Although the time for echocardiography was not documented, the total procedure time, which includes time to perform echocardiography, serves as an adequate surrogate when comparing procedural time between the 2 groups. Ultrasound evaluation of femoral vessels was performed only in patients with clinically apparent arterial injury, suggesting that true rates of arterial injury are likely underestimated. However, this is believed to adequately capture clinically important vascular complications. As this is a retrospective study, criteria for assessment of ductal spasm or adequacy of angiography were not defined. While ductal spasm can affect procedural success or increase risk of complications, successful PDA closure in this study supports the notion that clinically significant ductal spasm and/or inadequate angiography did not occur.
